# Cost-effectiveness of immunotherapies for advanced squamous non-small cell lung cancer: a systematic review

**DOI:** 10.1186/s12885-024-12043-w

**Published:** 2024-03-06

**Authors:** Minyu Cheng, Yanfei Shao, Li Li, Menglao Jiang, Zhouye Song

**Affiliations:** 1 Department of Pharmacy, Zhejiang Hospital, 310013 Hangzhou, China; 2 Department of Pharmacy, Zhejiang Provincial Peopleʼs Hospital, 310014 Hangzhou, China; 3 Zhejiang Center of Drug and Cosmetics Evaluation, 310000 Hangzhou, Zhejiang China

**Keywords:** Cost-effectiveness, Immunotherapy, Non-small cell lung cancer, Nivolumab, Pembrolizumab, Sintilimab, Toripalimab, Camrelizumab, Sugemalimab

## Abstract

**Background:**

There are differences in the pharmacoeconomics of Immune checkpoint blocking (ICB) therapies for the treatment of lung squamous cell carcinoma (LSCC). However, no corresponding review studies have fully discussed the cost-effectiveness of ICBs in treating LSCC. The aim of this paper is to systematically review and evaluate all available pharmacoeconomic studies of ICBs for LSCC.

**Method:**

The inclusion criteria were based on the population, intervention, comparator, outcomes, and study designs. An electronic search was conducted by June 2023, and the following databases were used: PubMed, EMBASE, Cochrane Library, and Web of Science. Search keywords included *‘Carcinoma’, Non-Small-Cell Lung’, ‘Immunotherapy’, and ‘Economics, Medical’*. The primary outcome was the cost-effectiveness analysis of ICB therapy in LSCC patients. Drummond Checklist was used to assess quality problems and possible bias in the study design of included pharmacoeconomic studies.

**Results:**

This review searched 15 articles on the economic evaluation of ICB treatment for LSCC. After a qualitative review of 15 studies, we concluded that nivolumab is more cost-effective as a monotherapy than chemotherapy alone. In the combination regimen, pembrolizumab combined with chemotherapy appears to be the most cost-effective option at present, but for Chinese payers with LSCC, locally developed treatments such as sintilimab or toripalimab in combination with chemotherapy are more cost-effective.

**Discussion:**

The inclusion of economic evaluation has heterogeneity in research design and outcomes, which can only support qualitative synthesis. Therefore, The results of this paper need to be treated with caution. For the Chinese market, instead of imported drugs, the possible cost-effectiveness of locally developed ICB therapies should be the focus of future research.

**Supplementary Information:**

The online version contains supplementary material available at 10.1186/s12885-024-12043-w.

## Background

Lung cancer is the most common cancer in the world and the most common cause of cancer death [1]. About half of patients with non-small cell lung cancer (NSCLC) are diagnosed with advanced stage [2]. Lung adenocarcinoma (LUAD) and lung squamous cell carcinoma (LSCC) are the most common clinical subtypes, with the latter accounting for about 30% of NSCLC patients [3, 4]. Meanwhile, more than half of patients with LSCC are over the age of 70 years, and age-related multi-organ decline changes the pharmacokinetics, which can increase the risk of local and systemic treatment complications [5, 6]. Although platinum dual chemotherapy is still the standard first-line treatment for advanced lung cancer patients whose tumors lack operable gene changes, it cannot be denied that chemotherapy alone has long reached a plateau of efficacy, and immunotherapy has changed the treatment regimen for some patients [7, 8].

Progression-free survival (PFS) and overall survival (OS) in LUAD patients are increasing with the deeper understanding of carcinogenic factors and the continuous development of targeted drugs, but in contrast, early studies have shown that the use of targeted drugs is associated with poor prognostic outcomes in patients with LSCC (grade 3 to 4 adverse events and even death were observed) [9–12].The Lung Master Protocol (Lung-MAP, S1400), based on next-generation gene sequencing technology, verified the efficacy of existing targeted drugs in LSCC patients through multiple sub-trials, and achieved an overall response rate of only 6-7% [11, 13, 14]. This changed after breakthroughs in the clinical translation of immunomodulatory antibodies, and immune checkpoint blocking (ICB) therapies, particularly those targeting the programmed death-1 pathway, have resulted in sustained immune efficacy, extended survival, and manageable adverse reactions in patients with NSCLC [15]. Currently, pembrolizumab and atezolizumab have been observed in randomized controlled trials with chemotherapy for longer OS and are approved by the U.S. Food and Drug Administration (FDA) as first-line agents for patients with advanced non-small cell lung cancer and PD-L1 expression on at least 50% of tumor cells [16, 17]. Moreover, Longer OS and PFS can be obtained from the pembrolizumab plus chemotherapy regimen for previously untreated patients with LSCC [18]. So far, ICB therapies including nivolumab [19], ipilimumab [20], sugemalimab [21], sintilimab [22], camrelizumab [23], tislelizumab [24], and cemiplimab [25] have been observed to significantly improve the prognosis of patients with LSCC. While the survival benefits these immunotherapies provide to patients with advanced LSCC are commendable, the financial strain and disease burden associated with the high price of ICB therapy cannot be ignored.

Multiple studies have demonstrated that pembrolizumab monotherapy is a cost-effective treatment option compared to chemotherapy for PD-L1-positive NSCLC patients [26–28] The incremental cost-effectiveness ratio for pembrolizumab monotherapy in the United States was $97,621/quality-adjusted life-year (QALY) in 2017 and $130,155/QALY in 2019 [26, 27]. In the cost-effectiveness analysis of the combination treatment regimen, there was considerable heterogeneity in the cost-benefit outcomes of nivolumab plus ipilimumab combined with or without chemotherapy [29–33] and pembrolizumab combined with chemotherapy [33–35] compared with platinum-doublet chemotherapy. In patients with advanced NSCLC characterized by either PD-L1 expression levels ≥ 50% or high TMB, Nivolumab + ipilimumab is more cost-effective as first-line treatment than chemotherapy, with ICER of $107,403.72 and $133,732.20, respectively [30]. Pembrolizumab plus chemotherapy produces ICERs close to or well below 3 times the U.S. GDP per capita threshold, and ICERs outcomes from current studies average around $100,000/QALY, which is considered a cost-effective treatment option [33, 34]; Other research contradicts this view [29, 31, 32, 35]. Model analysis based on the willingness to pay of American patients showed that compared with chemotherapy, the ICER of nivolumab plus Ipilimumab ranged from $202,275/QALY to $239,072 /QALY, and when the threshold was $150,000, the probability of being cost-effective was 2.6% [29, 31]. In addition, with ICER between $333,199 to $670,309.66 per QALY, the cost-effectiveness of atezolizumab, also a first-line treatment for LSCC, has been negated by both US and Chinese studies, and price reductions have been suggested [36–38]. Most of the published reviews in this area have targeted patients with non-small cell lung cancer [28] and have focused on a specific region [39] or comparison of two specific therapies [40]. Therefore, no studies have simultaneously compared the cost-effectiveness of all the immunotherapies in patients with LSCC. This study aimed to conduct a systematic review to summarize the cost-effectiveness of all these immunotherapies in patients with LSCC using the pooled analysis of the primary data on these ICB therapies.

## Methods

### Research design

The present systematic review was performed according to the PRISMA (Preferred Reporting Items for Systematic Reviews and Meta-analyses statement) [41]. The protocol for the present systematic review was officially registered on PROSPERO (CRD42023421278).

### Search strategy and data sources

We searched PubMed, EMBASE, Cochrane, and Web of Science with a pre-designed search strategy in June 2023 to retrieve all relevant clinical trials, using the MeSH terms *‘Carcinoma’, Non-Small-Cell Lung’, ‘Immunotherapy’, and ‘Economics, Medical’*, as well as relevant keywords. The detailed search strategy for all databases is reported in Supplementary Table 1. Besides, we searched all references in relevant articles and reviews to get other eligible studies, and we also retrieved articles by manual screening. Each study was assessed by two independent reviewers and disagreements were resolved by discussion with a third reviewer.

### Inclusion and exclusion criteria

Our selection criteria were generated based on the PICOS principle as follows.

### Inclusion criteria

P Patients were clinically diagnosed with squamous non-small cell lung cancer.
I Intervention groups received any immunotherapy;
C No restriction on the intervention of control groups;
O Incremental cost per QALY or ICER of immunotherapy and control should be provided;
S Cost-effectiveness analysis published in the English language.

### Exclusion criteria

A. Ineligible study design, such as case series, observational studies, commentary, and conference abstracts.
B. Essential data were absent from studies although emailed authors to obtain it.
C. Older duplicate reports published by the same team based on the same group of participants.
D. Studies included ineligible participants, such as participants with other cancer or not receive immunotherapy.
E. Cost-effectiveness analysis results not available.

### Data extaction

We used a pre-designed Excel spreadsheet to extract data for the included studies. Independent researchers worked in pairs to extract data, and inconsistencies were resolved by discussion or by having a third reviewer. Where there was unreported data in the studies, the authors were contacted for additional data; the rest of the data were publicly available as reported in the paper. The characteristics of the included studies are summarized as follows: name of the first author, year of publication, study country, study design, cost-effectiveness model, model developed with health states, participant number and diagnosis, administration design of intervention groups and control groups, and main conclusion.

### Quality assessment

Drummond Checklist was used to assess quality problems and possible bias in the study design of included pharmacoeconomic studies, inconsistencies were resolved by discussion or by having a third reviewer. The Drummond Checklist provides useful guidance applied to clarify the included studies with 10 answerable questions (yes, no, or not available), assuming the assessment result is strong, moderate, or weak [42].

## Results

### Results of study selection

**Fig. 1:**
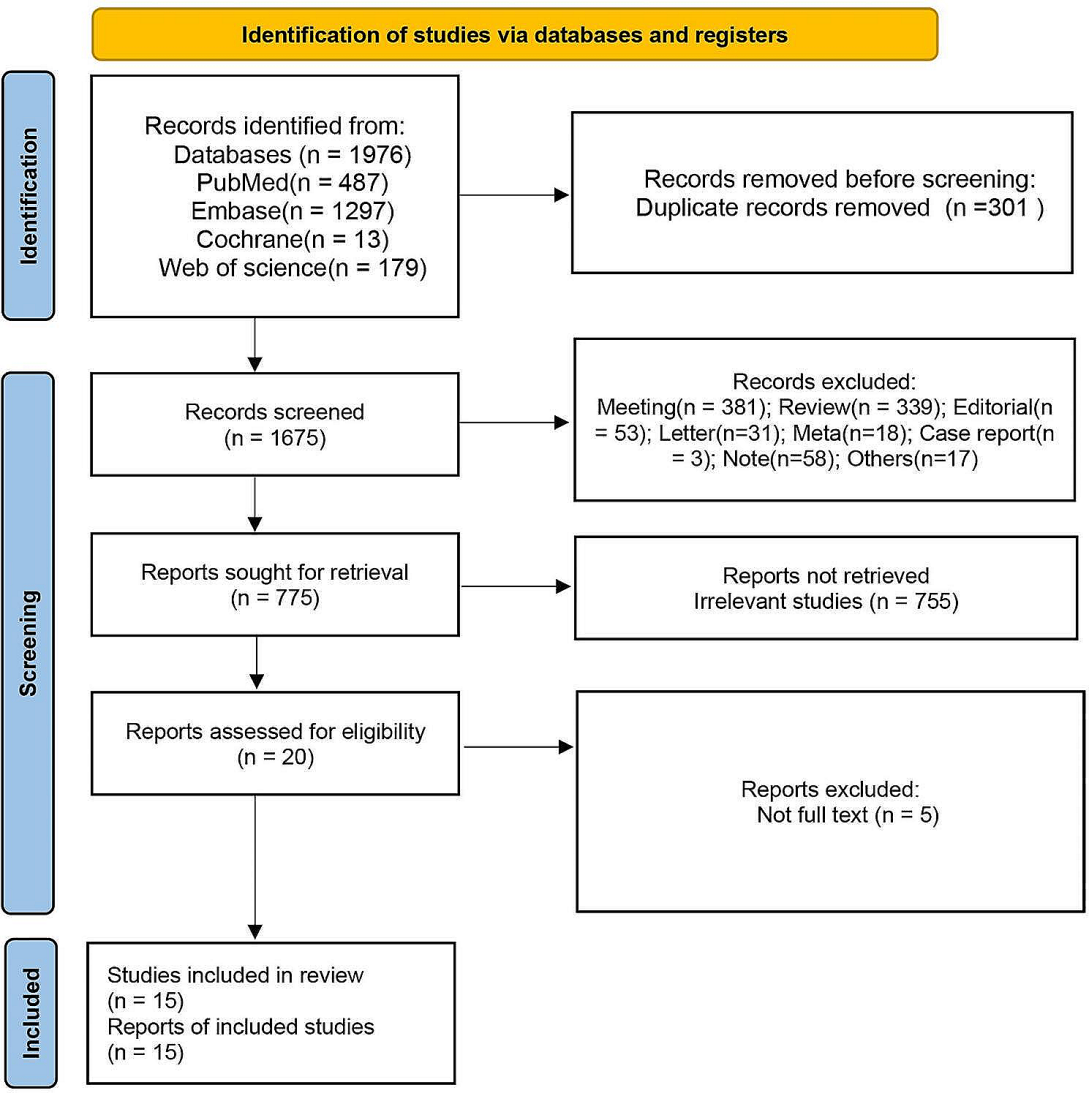
Flowchart of the study selection

In sum, 1976 articles were identified in electronic and manual searches. However, 301 articles were excluded for duplication. 900 records were excluded after reviewing the title and abstract, and we excluded 5 records after reviewing the full text of 20 articles. The exclusion reasons were full text not available. Finally, 15 articles [33, 43–56] were included in this systematic review (Fig. 1).

### Study characteristics

The basic characteristics of the 15 included full-text studies are shown in Table 1. Most of the studies were performed in China (n = 9), other studies were conducted in Canada (n = 2), Sweden (n = 1), Australia (n = 1), United Kingdom (n = 1), and America (n = 1). All included study designs were model-based cost-effectiveness analysis. The models used mainly include Cohort-based, partitioned survival model and Markov model. Two studies used both models to assess the cost-effectiveness of treatment options. Fourteen of the 15 studies selected progression-free (PF), progressed disease (PD), and death as health endpoints for model evaluation. Cheng et al. selected PFS, first disease progression, second disease progression, end-stage disease, and death as the health endpoints for model evaluation.

**Table 1:** Characteristics of the included studies

Study	Country	Study design	Mean age (years)	Male sex (%)	Model	Model Developed With Health States	Participant	Intervention	Comparison
Chaudhary 2021	Canada and Sweden	Model-Based	NR	NR	Cohort-based, partitioned survival model	progression-free (PF), progressed disease (PD), and death	advanced squamous NSCLC patients	The dosage of nivolumab applied was 240 mg every 2 weeks for Sweden and 480 mg every 4 weeks for Canada.	Docetaxel
Gao 2018	Australia	Model-Based	NR	NR	Both Markov and partition survival model	progression-free (PF), progressed disease (PD), and death.	260 patients with advanced or metastatic squamous nonsmallcell Lung cancer	Nivolumab 3 mg/kg, per 2 weeks	Docetaxel
Goeree 2016	Canada	Model-Based	NR	NR	Both Markov and partition survival model	progression-free (PF), progressed disease (PD), and death.	272 advanced squamous NSCLC patients who have failed one prior platinum doublet-based chemotherapy treatment.	Nivolumab	Docetaxel
Chouaid 2019	France	Model-Based	65	61.3	Cohort-based, partitioned survival model	progression-free (PF), progressed disease (PD), and death	55 adults, diagnosed with metastatic NSCLC	Pembrolizumab	Platinum agents
Rothwell 2021	UK	Model-Based	NR	NR	Cohort-based, partitioned survival model	progression-free (PF), progressed disease (PD), and death	Adults with advanced or metastatic squamous NSCLC after failure of prior platinum doublet-based chemotherapy	nivolumab 3 mg/kg every 2 weeks	docetaxel
Hu 2023	China	Model-Based	NR	NR	Cohort-based, partitioned survival model	progression-free (PF), progressed disease (PD), and death	advanced squamous NSCLC patients	Nivolumab	Docetaxel
Zhao 2023	China	Model-Based	NR	NR	Cohort-based, partitioned survival model	progression-free (PF), progressed disease (PD), and death	Chinese adults (aged ≥ 18 years) who had pathologically confirmed stage IIIB–IV wild-type sqNSCLC with unlimited PD-L1 expression.	paclitaxel and platinum combined with camrelizumab	cisplatin or carboplatin combined with gemcitabine, docetaxel, or paclitaxel
Cheng 2022	China	Model-Based	NR	NR	Markov model	PFS, first disease progression, second disease progression, end-stage disease, and death	patients with driver-negative advanced or metastatic sqNSCLC	First-line sintilimab	Second-line sintilimab
Insinga 2019	USA	Model-Based	NR	NR	Cohort-based, partitioned survival model	progression-free (PF), progressed disease (PD), and death	patients with squamous metastatic (stage 4) NSCLC tumor and are eligible for first-line systemic chemotherapy.	Pembrolizumab(200 mg, once every 3 weeks) plus chemotherapy	carboplatin and paclitaxel or nab-paclitaxel
Liu 2022	China	Model-Based	NR	NR	Markov model	progression-free (PF), progressed disease (PD), and death	squamous NSCLC patients	Pembrolizumab + chemotherapy	Pembrolizumab
Zhou 2023	China	Model-Based	NR	NR	Markov model	progression-free (PF), progressed disease (PD), and death	squamous NSCLC patients	toripalimab with chemotherapy	placebo with chemotherapy
Li 2022	China	Model-Based	NR	NR	Markov model	progression-free (PF), progressed disease (PD), and death	squamous NSCLC patients	sugemalimab(1,200 mg, once every 3 weeks) plus chemotherapy	placebo plus chemotherapy
Chen 2022	China	Model-Based	A: 61.48 B: 65.00 C: 65.00	A: 91.60 B: 81.40 C: 81.40	Cohort-based, partitioned survival model	progression-free (PF), progressed disease (PD), and death	stage III or IV squamous NSCLC patients	Sintilimab 200 mg every 3 weeks in combination with gemcitabine and either cisplatin or carboplatin for four cycles	Pembrolizumab 200 mg plus carboplatin and paclitaxel/nabpaclitaxel every 3 weeks
Shao 2022	China	Model-Based	NR	NR	Cohort-based, partitioned survival model	progression-free (PF), progressed disease (PD), and death	stage IIIB-IV sq-NSCLC patients	camrelizumab (200 mg) + carboplatin + paclitaxel	Placebo + carboplatin + paclitaxel
Zhang 2023	China	Model-Based	63	79.9	Cohort-based, partitioned survival model	progression-free (PF), progressed disease (PD), and death	those in the clinical trial in that all patients were treatment-naive, locally progressed or metastatic NSCLC	toripalimab 240 mg in combination with standard chemotherapy	placebo once per cycle in combination with standard chemotherapy

A: Sintilimab group (before adjustment); B: Pembrolizumab group; C: Sintilimab group (after adjustment); NR: Not reported

The target population of 9 studies was only patients with advanced or metastatic LSCC. In two of the included studies, the target population was restricted to failure of prior platinum doublet-based chemotherapy. The other two studies included patients with driver-negative advanced or metastatic LSCC. There was one study required squamous metastatic NSCLC patients eligible for first-line systemic chemotherapy. The remaining two studies included patients with a clinical diagnosis of metastatic NSCLC, and one of the studies required that the patients had not received any treatment. These two studies were included because patients with NSCLC were grouped according to whether they had squamous cancer or not, therefore the cost-effectiveness analysis data for the LSCC group was available.

All studies compared the cost-effectiveness of immunotherapy monotherapy or combination regimens with other interventions. In the intervention group, the immunotherapy regimen included nivolumab (n = 5), Pembrolizumab (n = 2), and sintilimab(n = 1). The remaining seven immunocombination regimens included camrelizumab (n = 2), pembrolizumab(n = 1), toripalimab(n = 2), sugemalimab(n = 1), and Sintilimab(n = 1) in combination with chemotherapy. As the control groups, five studies selected chemotherapy monotherapy using Docetaxel. Three studies selected the combination chemotherapy administration regimen, including Platinum agents, cisplatin or carboplatin combined with gemcitabine, docetaxel, or paclitaxel and carboplatin and paclitaxel or nab-paclitaxel. The aim of three included studies was to compare the cost-effectiveness of different immunotherapies, so the control group still chose the immune drugs. The remaining four studies used placebos as controls.

### Main conclusion of the included studies

Basic scenario results on cost-effectiveness between ICBs and other anticancer therapy are presented in Table 2. A total of 15 incremental costs, 4 incremental costs per life-year gained (LYG), 9 incremental cost per quality-adjusted life year (QALY), and 7 ICERs were available to compare ICBs with other anticancer drug groups (Table 2).

**Table 2:** Cost-Effectiveness analysis results of the included studies

Study	Main Conclusion
Chaudhary (2021)	Trial data demonstrated that nivolumab is associated with increased OS and response rates compared with docetaxel in patients with advanced pre-treated squamous, and suggested that nivolumab generates more favorable ICERs
Gao (2018)	The treatment with nivolumab cannot be considered cost-effective.
Goeree (2016)	For patients with advanced squamous NSCLC, nivolumab was found to have the highest expected per patient cost, but also higher LYs and QALYs compared to docetaxel.
Chouaid (2019)	Pembrolizumab appears cost-effective versus chemotherapy for first-line treatment of PD-L1positive (50%) metastatic NSCLC patients
Rothwell (2021)	Nivolumab versus docetaxel is cost effective for treating locally advanced/metastatic squamous NSCLC
Hu (2023)	Nivolumab yielded survival and quality-adjusted survival benefits at incremental cost versus docetaxel in aNSCLC.
Zhao (2023)	Paclitaxel and platinum combined with camrelizumab are the cost-effective treatment
Cheng (2022)	For Chinese patients with driver-negative advanced or metastatic sqNSCLC, reserving the use of sintilimab until the second-line represents a cost-effective treatment strategy compared with the first-line treatment.
Insinga (2019)	The addition of pembrolizumab to chemotherapy is projected to approximately double life expectancy, and can be a cost-effective first-line treatment for eligible metastatic squamous NSCLC patients for whom chemotherapy is currently administered.
Liu (2022)	For the squamous NSCLC patient population, the first-line Pembro + Chemo as a cost-effective treatment.
Zhou (2023)	Toripalimab plus chemotherapy was an optimal choice as first-line treatment.
Li (2022)	Sugemalimab plus chemotherapy was not cost-effective in comparison to placebo plus chemotherapy as first-line treatment for NSCLC, regardless of PD-L1 tumor expression level and pathological subtype.
Chen (2022)	Compared with pembrolizumab + chemotherapy, sintilimab + chemotherapy is more cost-effective for first-line treatment in Chinese patients with advanced or metastatic squamous NSCLC.
Shao (2022)	Camrelizumab plus chemotherapy was unlikely to be cost-effective compared with chemotherapy in the first line therapy of sq-NSCLC from a perspective of the Chinese healthcare system
Zhang (2023)	Toripalimab plus chemotherapy was cost-effective compared to chemotherapy for patients with advanced NSCLC in China.

Based on the main conclusions of the included studies, 9 studies model analysis results showed that the ICB intervention groups was cost-effective compared to the control groups, but 4 study results indicated that the ICB intervention group could not be considered cost-effective. Two other studies comparing different ICB therapies indicated that second-line sintilimab and sintilimab plus chemotherapy was the more cost-effective option compared to pembrolizumab or first-line sintilimab.

Specific analysis results were shown in Table 3 and the following systematic review section.

**Table 3:** Cost-effectiveness analysis results of the included studies

Study	Economic Study Design	Year to Which Costs Applied	Currency Used to Which Cost Applied	Cost of Study Intervention	Cost of Study Control	Incremental Cost	Total LYs of Intervention	Total LYs of Control	Total QALYs of Intervention	Total QALYs of Control	Incremental cost per LYG	Incremental cost per QALY	|ICER|
Chaudhary (2021)	Model-Based	2019	CAD and SEK	CAD 147,890 and SEK 736,478	CAD 51,439 and SEK 201,146	CAD 96,451 and SEK 535,333	CAD 1.77 and SEK 2.33	CAD 0.91 and SEK 1.29	CAD 1.29 and SEK 1.70	CAD 0.60 and SEK 0.95	CAD 112,921 and SEK 417,693	CAD 140,753 and SEK 568,895	NA
Gao (2018)	Model-Based	2017	USD	PS model: USD 137,935 and Markov model USD 100,236	PS model: USD 119,257 and Markov model USD 22,534	PS model: USD 118,678 and Markov model USD 77,702	PS model: 1.51 and Markov model 1.32	PS model: 0.86 and Markov model 0.91	PS model: 1.06 and Markov model 1.03	PS model: 0.86 and Markov model 0.68	NA	NA	PS model: 17,239 and Markov model 26,570
Goeree (2016)	Model-Based	2015	USD	PS model: USD 139,017 and Markov model USD 139,016	PS model: USD 38,849 and Markov model USD 38,812	PS model: USD 100,168 and Markov model USD 100,204	PS model: 1.69 and Markov model 1.68	PS model: 0.87 and Markov model 0.87	PS model: 1.24 and Markov model 1.23	PS model: 0.58 and Markov model 0.58	PS model: USD 121,905 and Markov model USD 122,834	PS model: USD 151,560 and Markov model USD 152,229	NA
Hu (2023)	Model-Based	2019	USD	USD 478,830	USD 264,477	USD 214,353	NA	NA	NA	NA	USD159,834	USD 190,919	207,388
Insinga (2019)	Model-Based	2018	USD	USD 231,209	USD 111,758	USD 119,451	3.4	1.76	2.8	1.41	USD72,725	USD 86,293	NA
Chen (2022)	Model-Based	2021	USD	USD 12,321	USD 36,371	USD24,050	1.84	1.74	0.99	1	NA	USD 1,314,280	NA
Cheng (2022)	Model-Based	2021	USD	USD 12,203	USD 14,045	USD 1842	NA	NA	1.37	1.52	NA	USD 12,694	NA
Chouaid (2019)	Model-Based	2017	EUR	EUR 125,261	EUR 63,229	EUR 62,032	2.14	1.21	1.57	0.83	NA	EUR 84,097	66,825
Liu (2022)	Model-Based	2021	USD	USD 153,892	USD 150,444	USD 3,449	NA	NA	NA	NA	NA	USD 15,613	NA
Rothwell (2021)	Model-Based	2015	GBP	GBP 46,711	GBP 15,430	GBP 31,281	NA	NA	1.39	0.51	NA	GBP 35,657	33,134
Shao (2022)	Model-Based	2021	USD	USD 19,165.08	USD 12,817.27	USD 6347.81	2.38	1.47	NA	NA	NA	NA	13571.68
Zhang (2023)	Model-Based	2021	USD	USD 45,268	USD 26,768	USD 18,501	NA	NA	1.4	0.83	NA	NA	32,237
Zhao (2023)	Model-Based	2022	USD	USD 19,026	USD 18,131	USD 895	2.513	2.219	1.603	1.283	NA	NA	NA
Zhou (2023)	Model-Based	2022	USD	USD 23,674	USD 11,367	USD 12,307	NA	NA	1.61	0.94	NA	NA	18,369
Li (2022)	Model-Based	2021	USD	USD 80,540.73	USD 24,726.85	USD 55,813.88	NA	NA	1.58	1	NA	NA	96,230.83

ICER: incremental cost-effectiveness ratios;QALY: quality-adjusted life year; LY, life-year; LYG, life-year gained; NSCLC, non-small cell lung cancer; USD, United States Dollar; CAD, Canadian Dollar; SEK, Swedish Krona; EUR, Euro; GBP, British Pound Sterling

### ICB monotherapy VS Chemo-based monotherapy

There were 5 studies that compared the cost-effectiveness of ICB monotherapy versus chemo-based monotherapy, and nivolumab and docetaxel were selected as the study intervention and control. Four studies confirmed the cost-effectiveness of nivolumab.

The results of four of the five studies comparing nivolumab and docetaxel suggest that nivolumab has a cost-effectiveness or advantage. Two of the studies based their judgments on ICERs. The Hu 2023 [49] study results showed an increase of RMB ¥207,388 (US $31,537) per QALY for nivolumab versus docetaxel ICER in squamous aNSCLC. Rothwell 2021 showed ICERs of £33,134 for nivolumab in patients with LSCC compared to docetaxel. The other two studies chose incremental cost per QALY as a measure of cost-effectiveness. Chaudhary 2021 [43] includes two cost-benefit analyses based on 5-year data from Canada or Sweden, with ICERs of $140,753 per QALY for the Canadian LSCC patients and SEK 568,895 per QALY for Sweden.

Gao 2018 [47] evaluated cost-effectiveness using partition survival (PS) and Markov models, respectively, and both showed that Nivolumab was associated with higher costs and benefits. The PS model showed an increase in the cost of treatment with nivolumab of $198,862 /QALY and $181,623 /LY. The ICER of nivolumab in Markov model is 220,029 AUD /QALY and 193,459 AUD /LY, respectively. Based on the willingness to pay a threshold of A $50,000 per QALY in Australia, the authors do not consider Nivolumab to be cost-effective.

### ICB monotherapy VS Chemo-based combination therapy

Two included studies compared the cost-effectiveness of pembrolizumab and platinum agents or pembrolizumab combined chemotherapy, respectively. Incremental cost per QALY was used as the evaluation index. Two studies had conflicting results on whether pembrolizumab was cost-effective compared to the control group.

In the study by Chouaid et al. [46], ICER for patients with LSCC was €66,825 /LY for pembrolizumab and €84,097 /QALY for platinum-based dual agents. Assuming a threshold of willingness to pay below €100,000 /QALY, pembrolizumab is cost-effective in first-line treatment of patients with PD-L1-positive (50%) metastatic NSCLC in France.

Liu 2021 [50] confirmed that compared to pembrolizumab monotherapy, pembrolizumab combined chemotherapy could add 0.22 QALY to life expectancy in patients with LSCC, with a corresponding marginal incremental cost of $3,449, and $15,613 /QALY of ICER.

### ICB-Chemo combination therapy VS chemo-based combination therapy

Two studies showed that ICB-Chemo combination therapy was cost-effective compared to Chemo-based combination therapy.

Insinga 2019 [34] shows that the pembrolizumab-chemo combination (P + C) group had 1.95 years more life expectancy than the chemo-based combination group (3.86 versus 1.91), resulting in an ICER of $86,293 /QALY. With ICER below $100,000/QALY as the maximum threshold, the P + C group is cost-effective. Meanwhile, with 2.513 LYs and 1.603 QALYs, Zhao 2023 [55] demonstrated camrelizumab plus platinum and paclitaxel chemotherapy as most cost-effective first-line choice.

### ICB-Chemo combination therapy VS placebo-chemo combination therapy

Four Chinese studies compared the cost-effectiveness of locally developed ICB-Chemo combination therapy and placebo-Chemo combination therapy, and the cost-effectiveness of two toripalimab plus chemotherapy studies was demonstrated. Two other studies based on sugemalimab and camrelizumab showed that the ICB group was not cost-effective.

For Chnese patients with LSCC, comparing toripalimab combination therapy with chemotherapy, Zhou 2023 [56] showed an ICER of $18,369/QALY (threshold US $37,653/QALY), a higher ICER was obtained by Zhang 2023 [54] $32,237/QALY (threshold value ($37,654/QALY). Toripalimab plus chemotherapy was confirmed as an optimal choice for LSCC first-line treatment.

In contrast, the Li 2022 [52] analysis showed that with $37,663/QALYs as the threshold, the ICER of sugemalimab-Chemo therapy compared with placebo-Chemo combination therapy was $96,230.83/QALYs. Shao 2022 [53] found that camrelizumab combined with chemotherapy increased by 0.47 QALYs and 0.91 LYs compared to chemotherapy, with a corresponding incremental cost of $6,347.81 and $13,572 /QALY for ICER. Camrelizumab combined with chemotherapy was not considered cost-effective in the Chinese medical system.

### ICB-based therapy VS ICB-based therapy

Two Chinese studies comparing the cost-effectiveness of different ICB regimens showed that local developed sintilimab as second-line treatment for LSCC and sintilimab plus chemotherapy were the more cost-effective regimens.

Results from Cheng 2022 [45] showed that sintilimab retained for second-line use had a higher efficacy and medical cost than first-line treatment (US $12,203 vs. US $14,045), with a corresponding ICER of $12,693 /QALY, which was cost-effective. Compared with pembrolizumab plus chemotherapy, sintilimab combined chemotherapy also been confirmed by Chen 2022 [44] as a lower lifetime cost, fewer QALYs cost-effective option, with ICER of $1,314,208/QALY.

### Study sites subgroup analysis

Nine of the 15 studies were conducted in China. In addition to the two ICB vs. ICB studies, 4 of the 7 studies (57.14%) concluded that ICB therapy was cost-effective, and 3 studies concluded that ICB therapy was not cost-effective. At the same time, four of the seven studies examined the cost-effectiveness of domestic ICBs compared with placebo, and three compared the cost-effectiveness of imported ICBs compared with chemotherapy. 50% of domestic ICB studies (n = 2) found ICBs to be cost-effective relative to placebo, and about 66% (n = 2) of imported ICB studies found ICBs to be cost-effective relative to chemotherapy.

There were 83% non-Chinese studies evaluated ICBs as cost-effective in patients with squamous NSCLC in the country. Two of the included studies were conducted in European countries, including the UK and France. Both studies compared the cost-effectiveness of ICB monotherapy and chemotherapy and showed that ICB therapy was cost-effective. Two studies examined the cost-effectiveness of ICB monotherapy or combination chemotherapy for payers in the Americas. Both of the results confirmed the cost-effectiveness of ICB monocular or combined chemotherapy. Only one study, from Australia, compared the cost-effectiveness of Nivolumab 3 mg/kg per 2 weeks with Docetaxel, and the model analysis showed that treatment with opdivo could not be considered cost-effective at a threshold of US $50,000 [47]. A multicentre cost-benefit analysis was performed on both Canadian and Swedish payers to compare the cost-effectiveness of nivolumab and docetaxel. Among payers of squamous NSCLC in Canada, the ICERs of nivolumab were CAN$140,753/QALY and in Swedish squamous patients, the ICERs were SEK568,895/QALY. This assessment led to the approval of nivolumab in Canada and Sweden for previously treated NSCLC patients [43].

The average threshold selected for the Chinese studies was $39,275.25 /QALY and the average threshold for the non-Chinese studies was $102,000 /QALY, which may be the reason for the lower proportion of Chinese studies that considered ICB therapy to be cost-effective.

### Study quality

Table 4 shows the methodological quality assessment results of the included studies. All included studies can be considered as strong quality evidence from the perspective of study design. Based on the 10 evaluation criteria of the Drummond Checklist, 13 of the 15 cost-effectiveness analyses were evaluated with perfect scores. The remaining two studies, Liu 2022 [50] and Shao 2022 [53], received a score of 9 for not providing a calculation method of the cost discount rate over time.

**Table 4:** Quality evaluation by Drummond checklist

Drummond Checklist/Study Authors	Was a Well-Defined Question Posed in an Answerable Form?	Was a Comprehensive Description of the Competing Alternatives Given?	Was the Effectiveness of the Program Established?	Were All the Important and Relevant Costs and Consequences for Each Alternative Identified?	Were Costs and Consequences Measured Accurately in Appropriate Physical Units?	Were Costs and Consequences Valued Credibly?	Were Costs and Consequences Adjusted for Differential Timing?	Was an Incremental Analysis of Costs and Consequences of Alternatives Performed?	Was Allowance Made for Uncertainty in the Estimates of Costs and Consequences?	Did the Presentation and Discussion of Study Results Include All Issues of Concern to Users?	Total
Chaudhary (2021)	Yes	Yes	Yes	Yes	Yes	Yes	Yes	Yes	Yes	Yes	10
Gao (2018)	Yes	Yes	Yes	Yes	Yes	Yes	Yes	Yes	Yes	Yes	10
Goeree (2016)	Yes	Yes	Yes	Yes	Yes	Yes	Yes	Yes	Yes	Yes	10
Hu (2023)	Yes	Yes	Yes	Yes	Yes	Yes	Yes	Yes	Yes	Yes	10
Insinga (2019)	Yes	Yes	Yes	Yes	Yes	Yes	Yes	Yes	Yes	Yes	10
Chen (2022)	Yes	Yes	Yes	Yes	Yes	Yes	Yes	Yes	Yes	Yes	10
Cheng (2022)	Yes	Yes	Yes	Yes	Yes	Yes	Yes	Yes	Yes	Yes	10
Chouaid (2019)	Yes	Yes	Yes	Yes	Yes	Yes	Yes	Yes	Yes	Yes	10
Liu (2022)	Yes	Yes	Yes	Yes	Yes	Yes	No	Yes	Yes	Yes	9
Rothwell (2021)	Yes	Yes	Yes	Yes	Yes	Yes	Yes	Yes	Yes	Yes	10
Shao (2022)	Yes	Yes	Yes	Yes	Yes	Yes	No	Yes	Yes	Yes	9
Zhang (2023)	Yes	Yes	Yes	Yes	Yes	Yes	Yes	Yes	Yes	Yes	10
Zhao (2023)	Yes	Yes	Yes	Yes	Yes	Yes	Yes	Yes	Yes	Yes	10
Zhou (2023)	Yes	Yes	Yes	Yes	Yes	Yes	Yes	Yes	Yes	Yes	10
Li (2022)	Yes	Yes	Yes	Yes	Yes	Yes	Yes	Yes	Yes	Yes	10

## Discussion

This review searched 15 articles published between the establishment of the database and June 2023 on the economic evaluation of ICB treatment for LSCC. The 15 studies included in this study compared the cost-effectiveness of ICB monotherapy vs. Chemo-based monotherapy, ICB monotherapy VS Chemo-based combination therapy, ICB-Chemo combination therapy VS Chemo-based combination therapy, ICB-Chemo combination therapy VS placebo-Chemo combination therapy, and ICB-based therapy VS ICB-based therapy in patients with LSCC. In the included full articles, more than 69% of the comparisons showed that ICB-based monotherapy or combination therapy was cost-effective or advantageous compared to chemotherapy monotherapy or combination therapy and placebo combination chemotherapy in patients with LSCC. At the same time, although 31% of studies concluded that ICB therapy was not cost-effective, these studies confirmed that ICB therapy resulted in higher costs and greater benefits (LYs and QALYs) compared to the control group. The reason for determining that it is not cost-effective depends mainly on the willingness-to-pay thresholds in specific countries (i.e., cost per QALY gained).

In the field of ICB monotherapy, current results show that nivolumab has the potential to offer significant cost benefits to patients compared to standard chemotherapy regimens. The likely reason is that nivolumab offers an unprecedented survival benefit compared to the poorly tolerated and moderately effective nature of current chemotherapy regimens. There were clinically and statistically significant improvements in OS observed by Checkmate 017 (HR 0.59; 95% ci 0.44–0.79; The observed 1-year survival rate was 42% in the opdivomab group and 24% in the docetaxel group [19]. In addition, Nivolumab was associated with a lower incidence of AE, and the study showed that fewer drug-related AE were reported in the Nivolumab group compared to the docetaxel group [19]. Significant efficacy and better prognosis, while increasing QALY and LY, reduce the cost of follow-up health maintenance and improve patients’ willingness to pay. For the reasons outlined above, although Gao2018’s assessment of nivolumab for patients with advanced or metastatic LSCC cannot be considered cost-effective based on the WTP/QALY thresholds commonly cited in Australia, given the unmet clinical needs of Australian patients, funding may be made available to the public through special arrangements to support clinical use of nivolumab [47].

Based on current evidence, pembrolizumab monotherapy is more cost-effective as first-line treatment in patients with LSCC than combination chemotherapy [46], and pembrolizumab + chemotherapy has been shown to be more cost-effective than Pembrolizumab alone [50]. Sensitivity analysis of model parameters showed that in addition to first-line pembrolizumab + chemotherapy vs. pembrolizumab quantitative measures such as hazard ratios and AE that reflect the efficacy and safety of cancer therapy, drug price also had a considerable impact on our cost-effectiveness results. Previous studies have also indicated that the factor most likely to reverse the results of cost-benefit analysis is the cost difference between two competing treatments [57, 58]. Deterministic sensitivity analysis (DSA) results from the Liu et al. [50] showed that for the LSCC patient population, pembrolizumab’s price per mg ranked first among all drugs in the DSA. However, the model still affirmed the cost-effectiveness of pembrolizumab + Chemo because of the inclusion of first-line treatment disruptions due to AE and decreased effectiveness due to AE in this analysis.

In addition to the imported immunotherapies mentioned above, the clinical trial data of domestic inhibitors in recent years are also expected. In terms of the clinical efficacy of LSCC, analysis of OS in patients with LSCC treated with toripalimab combination showed no significant difference compared to placebo, but a median OS increase of 3.4 years was observed (21 vs. 17.6) [59]. The antitumor effect of sintilimab in combination with platinum plus gemcitabine for squamous NSCLC was evaluated in ORIENT-12/NCT03629925. The results showed that the median PFS was 5.5 months in the cintizumab group and 4.9 months in the placebo group (P < 0.00001) [60]. GEMSTONE-302, a double-blind, randomized, phase 3 clinical trial results found sugemalimab versus placebo, in combination with platinum-based chemotherapy compared with the placebo group, progression-free survival was significantly longer in the sugemalimab group (median 7.8 months [95% CI 6.9-9·0] vs. 4.9 months [4.7-5.0]; stratified hazard ratio [HR] 0·50 [95% CI 0.39–0.64], p < 0.0001) [21]. Results from the Phase 3 double-blind randomized controlled trial of camrelizumab plus chemotherapy (NCT03668496) showed a significant extension of PFS in patients with LSCC (median, 8.5 months vs. 4.9 months; P < 0.0001) [61].

At present, the cost-effectiveness studies of toripalimab, sugemalimab, and camrelizumab combined chemotherapy are still in a relatively preliminary stage, and the control group is placebo combined chemotherapy. Among these, sintilimab in combination with chemotherapy may be the most promising option based on current evidence. Compared with chemotherapy alone, the ICER of toripalimab plus chemotherapy was $32,237 /QALY, which was lower than Chinese WTP threshold ($37,654 /QALY). The health utility value of progression-free survival, the price of topalizumab and the cost of the best supportive treatment were significant factors influencing ICER [44]. Zhou 2023 mentioned in her study that previous studies based on imported inhibitors in China have not achieved satisfactory cost-effectiveness. However, compared to imported drugs, Chinese ontologic developed inhibitors such as camrelizumab, sintilimab and toripalimab achieve greater accessibility and cost-effectiveness at a lower price while balancing efficacy [56]. The study included in this review by Zhao et al. (2023) also confirmed the cost-effectiveness of camrelizumab in combination with chemotherapy in the treatment of patients with metastatic LSCC [55]. Similarly, although the amount of supporting evidence is limited, Chen 2022 [44] also showed that it is not pembrolizumab + chemotherapy that is more cost-effective for Chinese payers, but the locally developed regimen of sintilimab + chemotherapy [44]. The cost-benefit analysis depends heavily on the WTP threshold. While thresholds vary from country to country, reducing the cost of new drugs, for example through local research and development, is the most fundamental way to increase patient benefits and promote new drugs.

### Limitation

This systematic review incorporates most of the available literature and uses the Drummond checklist criteria for quality assessment, but some potential limitations remain. First of all, the language of the included study was limited to English, and the results of the cost-benefit analysis were significantly affected by regions, which may lead to insufficient comprehensive review results. Second, the conference abstracts that appear more frequently in cancer studies are excluded, so some of the most recent analytical results may be missed. Therefore, the conclusions given in this paper should be treated with caution. Third, the included economic assessment has heterogeneity in research design, such as model, viewpoint, target population and time range, which does not support quantitative synthesis of analysis results, and this study only makes a qualitative summary of evidence. Fourthly, our study primarily focused on LSCC and might not fully encapsulate the cost-effectiveness landscape for LUAD or the broader spectrum of NSCLC subtypes. Further research specifically targeting LUAD and other NSCLC subtypes is warranted to provide a more comprehensive understanding of the cost-effectiveness of treatment options across the NSCLC spectrum. Finally, although 9 of the included studies were from China, and certain conclusions were drawn based on the review analysis, the intervention methods used in the studies were still relatively scattered, and no uniform answer could be reached on the best cost-effective choice of ICB for the treatment of LSCC. However, it cannot be denied that the future prospect of cost-effectiveness research on locally developed ICB drugs is worth looking forward to. Future research should incorporate a broader array of studies from different regions.

## Conclusion

This systematic review brings together as many pharmacoeconomic studies on ICB treatment of LSCC as possible to date. After a qualitative review of 15 studies, we concluded that nivolumab is more cost-effective as a monotherapy than chemotherapy alone. In the combination regimen, pembrolizumab combined chemotherapy appears to be the most cost-effective option at present, but for Chinese payers with LSCC, locally developed treatments such as sintilimab or toripalimab in combination with chemotherapy are more cost-effective.

### Electronic supplementary material

Below is the link to the electronic supplementary material.

Supplementary Material 1. Search strategy

## Data Availability

All data generated or analyzed during this study are included in this published article and its supplementary information files.

## Abbreviations

ICB: Immune checkpoint blocking
LSCC: lung squamous cell carcinoma
PFS: Progression-free survival
OS: overall survival
FDA: Food and Drug Administration
QALY: quality-adjusted life year
LYG: life-year gained

## References

[1] Brown S, Banfill K, Aznar MC, Whitehurst P, Faivre Finn C (2019). The evolving role of radiotherapy in non-small cell lung cancer. Br J Radiol.

[2] Russo AE, Priolo D, Antonelli G, Libra M, McCubrey JA, Ferraù F (2017). Bevacizumab in the treatment of NSCLC: patient selection and perspectives. Lung Cancer (Auckl).

[3] Niu Z, Jin R, Zhang Y, Li H (2022). Signaling pathways and targeted therapies in lung squamous cell carcinoma: mechanisms and clinical trials. Signal Transduct Target Ther.

[4] Herbst RS, Morgensztern D, Boshoff C (2018). The biology and management of non-small cell lung cancer. Nature.

[5] Sawhney R, Sehl M, Naeim A (2005). Physiologic aspects of aging: impact on cancer management and decision making, part I. Cancer J.

[6] Sehl M, Sawhney R, Naeim A (2005). Physiologic aspects of aging: impact on cancer management and decision making, part II. Cancer J.

[7] Baxevanos P, Mountzios G (2018). Novel chemotherapy regimens for advanced lung cancer: have we reached a plateau?. Ann Transl Med.

[8] Low JL, Walsh RJ, Ang Y, Chan G, Soo RA (2019). The evolving immuno-oncology landscape in advanced lung cancer: first-line treatment of non-small cell lung cancer. Ther Adv Med Oncol.

[9] Paik PK, Shen R, Berger MF, Ferry D, Soria JC, Mathewson A (2017). A phase ib open-label Multicenter Study of AZD4547 in patients with advanced squamous cell lung cancers. Clin Cancer Res.

[10] Nogova L, Sequist LV, Perez Garcia JM, Andre F, Delord JP, Hidalgo M (2017). Evaluation of BGJ398, a fibroblast growth factor receptor 1–3 kinase inhibitor, in patients with Advanced Solid tumors Harboring genetic alterations in fibroblast growth factor receptors: results of a global phase I, dose-escalation and dose-expansion study. J Clin Oncol.

[11] Aggarwal C, Redman MW, Lara PN, Borghaei H, Hoffman P, Bradley JD (2019). SWOG S1400D (NCT02965378), a phase II study of the fibroblast growth factor receptor inhibitor AZD4547 in previously treated patients with fibroblast growth factor pathway-activated stage IV squamous cell lung Cancer (Lung-MAP substudy). J Thorac Oncol.

[12] Langer CJ, Redman MW, Wade JL, IIIrd, Aggarwal C, Bradley JD, Crawford J (2019). SWOG S1400B (NCT02785913), a phase II study of GDC-0032 (Taselisib) for previously treated PI3K-Positive patients with stage IV squamous cell lung Cancer (Lung-MAP sub-study). J Thorac Oncol.

[13] Herbst RS, Gandara DR, Hirsch FR, Redman MW, LeBlanc M, Mack PC (2015). Lung Master Protocol (Lung-MAP)-A biomarker-driven protocol for accelerating development of therapies for squamous cell Lung Cancer: SWOG S1400. Clin Cancer Res.

[14] Edelman MJ, Redman MW, Albain KS, McGary EC, Rafique NM, Petro D (2019). SWOG S1400C (NCT02154490)-A phase II study of Palbociclib for previously treated cell cycle gene alteration-positive patients with stage IV squamous cell lung Cancer (Lung-MAP substudy). J Thorac Oncol.

[15] Forde PM, Kelly RJ, Brahmer JR (2014). New strategies in lung cancer: translating immunotherapy into clinical practice. Clin Cancer Res.

[16] Reck M, Rodríguez-Abreu D, Robinson AG, Hui R, Csőszi T, Fülöp A (2016). Pembrolizumab versus Chemotherapy for PD-L1-Positive non-small-cell Lung Cancer. N Engl J Med.

[17] Herbst RS, Giaccone G, de Marinis F, Reinmuth N, Vergnenegre A, Barrios CH (2020). Atezolizumab for First-Line treatment of PD-L1-Selected patients with NSCLC. N Engl J Med.

[18] Paz-Ares L, Luft A, Vicente D, Tafreshi A, Gümüş M, Mazières J (2018). Pembrolizumab plus Chemotherapy for squamous non-small-cell Lung Cancer. N Engl J Med.

[19] Brahmer J, Reckamp KL, Baas P, Crinò L, Eberhardt WE, Poddubskaya E (2015). Nivolumab versus Docetaxel in Advanced squamous-cell non-small-cell Lung Cancer. N Engl J Med.

[20] Govindan R, Szczesna A, Ahn MJ, Schneider CP, Gonzalez Mella PF, Barlesi F (2017). Phase III trial of Ipilimumab Combined with Paclitaxel and Carboplatin in Advanced squamous non-small-cell Lung Cancer. J Clin Oncol.

[21] Zhou C, Wang Z, Sun Y, Cao L, Ma Z, Wu R (2022). Sugemalimab versus placebo, in combination with platinum-based chemotherapy, as first-line treatment of metastatic non-small-cell lung cancer (GEMSTONE-302): interim and final analyses of a double-blind, randomised, phase 3 clinical trial. Lancet Oncol.

[22] Shi Y, Wu L, Yu X, Xing P, Wang Y, Zhou J (2022). Sintilimab versus Docetaxel as second-line treatment in advanced or metastatic squamous non-small-cell lung cancer: an open-label, randomized controlled phase 3 trial (ORIENT-3). Cancer Commun (Lond).

[23] Zhou C, Chen G, Huang Y, Zhou J, Lin L, Feng J (2021). Camrelizumab plus carboplatin and pemetrexed versus chemotherapy alone in chemotherapy-naive patients with advanced non-squamous non-small-cell lung cancer (CameL): a randomised, open-label, multicentre, phase 3 trial. Lancet Respir Med.

[24] Zhou C, Huang D, Fan Y, Yu X, Liu Y, Shu Y (2023). Tislelizumab Versus Docetaxel in patients with previously treated Advanced NSCLC (RATIONALE-303): a phase 3, Open-Label, Randomized Controlled Trial. J Thorac Oncol.

[25] Sezer A, Kilickap S, Gümüş M, Bondarenko I, Özgüroğlu M, Gogishvili M (2021). Cemiplimab monotherapy for first-line treatment of advanced non-small-cell lung cancer with PD-L1 of at least 50%: a multicentre, open-label, global, phase 3, randomised, controlled trial. Lancet.

[26] Huang M, Lopes GL, Insinga RP, Burke T, Ejzykowicz F, Zhang Y (2019). Cost-effectiveness of pembrolizumab versus chemotherapy as first-line treatment in PD-L1-positive advanced non-small-cell lung cancer in the USA. Immunotherapy.

[27] Huang M, Lou Y, Pellissier J, Burke T, Liu FX, Xu R (2017). Cost effectiveness of Pembrolizumab vs. Standard-of-care chemotherapy as first-line treatment for metastatic NSCLC that expresses high levels of PD-L1 in the United States. PharmacoEconomics.

[28] Ding H, Xin W, Tong Y, Sun J, Xu G, Ye Z (2020). Cost effectiveness of immune checkpoint inhibitors for treatment of non-small cell lung cancer: a systematic review. PLoS ONE.

[29] Yang SC, Kunst N, Gross CP, Wang JD, Su WC, Wang SY (2021). Cost-effectiveness of Nivolumab Plus Ipilimumab with and without chemotherapy for Advanced Non-small Cell Lung Cancer. Front Oncol.

[30] Hu H, She L, Liao M, Shi Y, Yao L, Ding D (2020). Cost-effectiveness analysis of Nivolumab plus Ipilimumab vs. Chemotherapy as First-Line Therapy in Advanced Non-small Cell Lung Cancer. Front Oncol.

[31] Li J, Zhang T, Xu Y, Lu P, Zhu J, Liang W (2020). Cost-effectiveness analysis of nivolumab plus ipilimumab versus chemotherapy as first-line treatment in advanced NSCLC. Immunotherapy.

[32] Peng Y, Zeng X, Peng L, Liu Q, Yi L, Luo X (2021). Cost-effectiveness of Nivolumab plus Ipilimumab Combined with two cycles of Chemotherapy as First-Line treatment in Advanced Non-small Cell Lung Cancer. Adv Ther.

[33] Insinga RP, Vanness DJ, Feliciano JL, Vandormael K, Traore S, Burke T (2018). Cost-effectiveness of pembrolizumab in combination with chemotherapy in the 1st line treatment of non-squamous NSCLC in the US. J Med Econ.

[34] Insinga RP, Vanness DJ, Feliciano JL, Vandormael K, Traore S, Ejzykowicz F (2019). Cost-effectiveness of pembrolizumab in combination with chemotherapy versus chemotherapy and pembrolizumab monotherapy in the first-line treatment of squamous non-small-cell lung cancer in the US. Curr Med Res Opin.

[35] Zeng X, Wan X, Peng L, Peng Y, Ma F, Liu Q (2019). Cost-effectiveness analysis of pembrolizumab plus chemotherapy for previously untreated metastatic non-small cell lung cancer in the USA. BMJ Open.

[36] Criss SD, Mooradian MJ, Watson TR, Gainor JF, Reynolds KL, Kong CY (2019). Cost-effectiveness of Atezolizumab Combination Therapy for First-Line treatment of metastatic nonsquamous non-small cell Lung Cancer in the United States. JAMA Netw Open.

[37] Lin S, Luo S, Zhong L, Lai S, Zeng D, Rao X (2020). Cost-effectiveness of atezolizumab plus chemotherapy for advanced non-small-cell lung cancer. Int J Clin Pharm.

[38] Li LY, Wang H, Chen X, Li WQ, Cui JW (2019). First-line atezolizumab plus chemotherapy in treatment of extensive small cell lung cancer: a cost-effectiveness analysis from China. Chin Med J (Engl).

[39] Yu A, Huang E, Abe M, An K, Park SK, Park C (2021). Cost-effectiveness analyses of targeted therapy and immunotherapy for advanced non-small cell lung cancer in the United States: a systematic review. Expert Rev Pharmacoecon Outcomes Res.

[40] Liu S, Jiang N, Dou L, Li S (2023). Cost-effectiveness analysis of serplulimab plus chemotherapy in the first-line treatment for PD-L1-positive esophageal squamous cell carcinoma in China. Front Immunol.

[41] Page MJ, McKenzie JE, Bossuyt PM, Boutron I, Hoffmann TC, Mulrow CD (2021). The PRISMA 2020 statement: an updated guideline for reporting systematic reviews. BMJ.

[42] Edwards DJCaPRT. A Guide To Health Economics for those Working in Public Health a concise desktop handbook. Public Health Wales. 2016.

[43] Chaudhary MA, Holmberg C, Lakhdari K, Smare C, Theriou C, Dale P (2021). Cost-effectiveness of nivolumab in squamous and non-squamous non-small cell lung cancer in Canada and Sweden: an update with 5-year data. J Med Econ.

[44] Chen P, Wang X, Zhu S, Li H, Rui M, Wang Y (2022). Economic evaluation of sintilimab plus chemotherapy vs. pembrolizumab plus chemotherapy for the treatment of first-line advanced or metastatic squamous NSCLC. Front Public Health.

[45] Cheng R, Zhou Z, Liu Q (2023). Cost-effectiveness of first-line versus second-line use of domestic anti-PD-1 antibody sintilimab in Chinese patients with advanced or metastatic squamous non-small cell lung cancer. Cancer Med.

[46] Chouaid C, Bensimon L, Clay E, Millier A, Levy-Bachelot L, Huang M (2019). Cost-effectiveness analysis of pembrolizumab versus standard-of-care chemotherapy for first-line treatment of PD-L1 positive (> 50%) metastatic squamous and non-squamous non-small cell lung cancer in France. Lung Cancer.

[47] Gao L, Li SC (2019). Modelled Economic evaluation of Nivolumab for the treatment of Second-Line Advanced or metastatic squamous non-small-cell Lung Cancer in Australia using both partition survival and Markov Models. Appl Health Econ Health Policy.

[48] Goeree R, Villeneuve J, Goeree J, Penrod JR, Orsini L, Tahami Monfared AA (2016). Economic evaluation of nivolumab for the treatment of second-line advanced squamous NSCLC in Canada: a comparison of modeling approaches to estimate and extrapolate survival outcomes. J Med Econ.

[49] Hu S, Tang Z, Harrison JP, Hertel N, Penrod JR, May JR (2023). Economic evaluation of Nivolumab Versus Docetaxel for the Treatment of Advanced Squamous and non-squamous non-small cell Lung Cancer after prior chemotherapy in China. Pharmacoecon Open.

[50] Liu Q, Zhou Z, Luo X, Yi L, Peng L, Wan X (2021). Cost-effectiveness of Pembrolizumab Plus Chemotherapy Versus Pembrolizumab Monotherapy in metastatic non-squamous and squamous NSCLC patients with PD-L1 expression ≥ 50. Front Pharmacol.

[51] Rothwell B, Kiff C, Ling C, Brodtkorb TH (2021). Cost effectiveness of Nivolumab in patients with Advanced, previously treated squamous and non-squamous non-small-cell Lung Cancer in England. Pharmacoecon Open.

[52] Li W, Wan L (2022). Cost-effectiveness analysis of sugemalimab vs. placebo, in combination with chemotherapy, for treatment of first-line metastatic NSCLC in China. Front Public Health.

[53] Shao T, Ren Y, Zhao M, Tang W (2022). Cost-effectiveness analysis of camrelizumab plus chemotherapy as first-line treatment for advanced squamous NSCLC in China. Front Public Health.

[54] Zhang M, Xu K, Lin Y, Zhou C, Bao Y, Zhang L (2023). Cost-effectiveness analysis of toripalimab plus chemotherapy versus chemotherapy alone for advanced non-small cell lung cancer in China. Front Immunol.

[55] Zhao M, Shao T, Chi Z, Tang W (2023). Effectiveness and cost-effectiveness analysis of 11 treatment paths, seven first-line and three second-line treatments for Chinese patients with advanced wild-type squamous non-small cell lung cancer: a sequential model. Front Public Health.

[56] Zhou K, Shu P, Zheng H, Li Q (2023). Cost-effectiveness analysis of toripalimab plus chemotherapy as the first-line treatment in patients with advanced non-small cell lung cancer (NSCLC) without EGFR or ALK driver mutations from the Chinese perspective. Front Pharmacol.

[57] Liu Q, Luo X, Yi L, Zeng X, Tan C (2021). First-line chemo-immunotherapy for extensive-stage small-cell lung Cancer: a United States-based cost-effectiveness analysis. Front Oncol.

[58] Liu Q, Luo X, Peng L, Yi L, Wan X, Zeng X (2020). Nivolumab Versus Docetaxel for previously treated Advanced Non-small Cell Lung Cancer in China: a cost-effectiveness analysis. Clin Drug Investig.

[59] Wang Z, Wu L, Li B, Cheng Y, Li X, Wang X (2023). Toripalimab Plus Chemotherapy for patients with treatment-naive Advanced Non-small-cell Lung Cancer: a Multicenter Randomized Phase III Trial (CHOICE-01). J Clin Oncol.

[60] Zhou C, Wu L, Fan Y, Wang Z, Liu L, Chen G (2021). Sintilimab Plus Platinum and Gemcitabine as First-Line treatment for Advanced or metastatic squamous NSCLC: results from a Randomized, Double-Blind, phase 3 trial (ORIENT-12). J Thorac Oncol.

[61] Ren S, Chen J, Xu X, Jiang T, Cheng Y, Chen G (2022). Camrelizumab Plus Carboplatin and Paclitaxel as First-Line treatment for advanced squamous NSCLC (CameL-Sq): a phase 3 trial. J Thorac Oncol.

