# Supplementary material for: Cost-effectiveness of immunotherapies for advanced squamous non-small cell lung cancer: a systematic review
Source: BMC Cancer. 2024 Mar 6;24:312. doi: 10.1186/s12885-024-12043-w (PMC10916025; doi:10.1186/s12885-024-12043-w)
Supplement: Supplementary file 1 — Supplementary Material 1. Search strategy [file 12885_2024_12043_MOESM1_ESM.docx]

**Supplementary table 1 search strategy**

**Search strategy of PubMed**

| NO. | Search Details | Results |
| --- | --- | --- |
| #8 | (#1 OR #4) AND (#2 OR #5 OR #7) AND (#3 OR #6) | 487 |
| #7 | ((((((((((((((Pembrolizumab) OR (Keytruda)) OR (Nivolumab)) OR (Cemiplimab)) OR (Durvalumab)) OR (Atezolizumab)) OR (Ipilimumab)) OR (Camrelizumab)) OR (Sintilimab)) OR (Tislelizumab)) OR (Sugemalimab)) OR (Serplulimab)) OR (Tiragolumab)) OR (Toripalimab)) OR (Tremelimumab) | 21,221 |
| #6 | (((((clinical economics) OR (dental economics)) OR (hospital economics)) OR (medical economics)) OR (nursing economics)) OR (health economics) | 1,864,765 |
| #5 | (((((((((((((((((((biologic response modifier therapy) OR (biological response modifier therapy)) OR (BRM therapy)) OR (immune therapy)) OR (immunogenic therapy)) OR (immunoglobulin therapy)) OR (immunological therapy)) OR (immunological treatment)) OR (immunomodulant therapy)) OR (immunomodulary therapy)) OR (immunomodulating therapy)) OR (immunomodulation therapy)) OR (immunomodulative therapy)) OR (immunomodulator therapy)) OR (immunomodulatory intervention)) OR (immunomodulatory therapy)) OR (immunomoduling therapy)) OR (immunomodurating therapy)) OR (Immunotherapies)) OR (Immunotherapy) | 2,091,200 |
| #4 | ((((((((((((((((((((bronchial non small cell cancer) OR (bronchial non small cell carcinoma)) OR (lung non small cell cancer)) OR (lung non small cell carcinoma)) OR (non oat cell lung cancer)) OR (non small cell bronchial cancer)) OR (non small cell lung cancer)) OR (Non Small Cell Lung Carcinoma)) OR (non small cell pulmonary cancer)) OR (non small cell pulmonary carcinoma)) OR (non squamous NSCLC)) OR (non-oat cell lung cancer)) OR (nonsmall cell carcinoma of the lung)) OR (Nonsmall Cell Lung Cancer)) OR (Non-Small Cell Lung Cancer)) OR (nonsmall cell lung carcinoma)) OR (Non-Small Cell Lung Carcinoma)) OR (Non-Small-Cell Lung Carcinoma)) OR (Non-Small-Cell Lung Carcinomas)) OR (pulmonary non small cell cancer)) OR (pulmonary non small cell carcinoma) | 104,127 |
| #3 | "Economics, Medical"[Mesh] | 14,392 |
| #2 | "Immunotherapy"[Mesh] | 331,915 |
| #1 | "Carcinoma, Non-Small-Cell Lung"[Mesh] | 70,108 |

**Search strategy of EMBASE**

| No. | Query | Results |
| --- | --- | --- |
| #8 | (#1 OR #2) AND (#3 OR #4 OR #7) AND (#5 OR #6) | 1297 |
| #7 | 'pembrolizumab':ti,ab,kw OR 'keytruda':ti,ab,kw OR 'nivolumab':ti,ab,kw OR 'cemiplimab':ti,ab,kw OR 'durvalumab':ti,ab,kw OR 'atezolizumab':ti,ab,kw OR 'ipilimumab':ti,ab,kw OR 'camrelizumab':ti,ab,kw OR 'sintilimab':ti,ab,kw OR 'tislelizumab':ti,ab,kw OR 'sugemalimab':ti,ab,kw OR 'serplulimab':ti,ab,kw OR 'tiragolumab':ti,ab,kw OR 'toripalimab':ti,ab,kw OR 'tremelimumab':ti,ab,kw | 43824 |
| #6 | 'clinical economics':ti,ab,kw OR 'dental economics':ti,ab,kw OR 'hospital economics':ti,ab,kw OR 'medical economics':ti,ab,kw OR 'nursing economics':ti,ab,kw OR 'health economics':ti,ab,kw | 10935 |
| #5 | 'health economics'/exp | 1038811 |
| #4 | 'biologic response modifier therapy':ti,ab,kw OR 'biological response modifier therapy':ti,ab,kw OR 'brm therapy':ti,ab,kw OR 'immune therapy':ti,ab,kw OR 'immunogenic therapy':ti,ab,kw OR 'immunoglobulin therapy':ti,ab,kw OR 'immunological therapy':ti,ab,kw OR 'immunological treatment':ti,ab,kw OR 'immunomodulant therapy':ti,ab,kw OR 'immunomodulary therapy':ti,ab,kw OR 'immunomodulating therapy':ti,ab,kw OR 'immunomodulation therapy':ti,ab,kw OR 'immunomodulative therapy':ti,ab,kw OR 'immunomodulator therapy':ti,ab,kw OR 'immunomodulatory intervention':ti,ab,kw OR 'immunomodulatory therapy':ti,ab,kw OR 'immunomoduling therapy':ti,ab,kw OR 'immunomodurating therapy':ti,ab,kw OR 'immunotherapies':ti,ab,kw OR 'immunotherapy':ti,ab,kw | 215348 |
| #3 | 'immunotherapy'/exp | 306616 |
| #2 | 'bronchial non small cell cancer':ti,ab,kw OR 'bronchial non small cell carcinoma':ti,ab,kw OR 'lung non small cell cancer':ti,ab,kw OR 'lung non small cell carcinoma':ti,ab,kw OR 'non oat cell lung cancer':ti,ab,kw OR 'non small cell bronchial cancer':ti,ab,kw OR 'non small cell lung cancer':ti,ab,kw OR 'non small cell lung carcinoma':ti,ab,kw OR 'non small cell pulmonary cancer':ti,ab,kw OR 'non small cell pulmonary carcinoma':ti,ab,kw OR 'non squamous nsclc':ti,ab,kw OR 'non-oat cell lung cancer':ti,ab,kw OR 'nonsmall cell carcinoma of the lung':ti,ab,kw OR 'nonsmall cell lung cancer':ti,ab,kw OR 'non-small cell lung cancer':ti,ab,kw OR 'nonsmall cell lung carcinoma':ti,ab,kw OR 'non-small cell lung carcinoma':ti,ab,kw OR 'non-small-cell lung carcinoma':ti,ab,kw OR 'non-small-cell lung carcinomas':ti,ab,kw OR 'pulmonary non small cell cancer':ti,ab,kw OR 'pulmonary non small cell carcinoma':ti,ab,kw | 131979 |
| #1 | 'non small cell lung cancer'/exp | 209774 |

**Search strategy of Cochrane Library**

| NO. | Search deatiles | Hits |
| --- | --- | --- |
| #1 | MeSH descriptor: [Carcinoma, Non-Small-Cell Lung] explode all trees | 5751 |
| #2 | MeSH descriptor: [Immunotherapy] explode all trees | 11696 |
| #3 | MeSH descriptor: [Economics, Medical] explode all trees | 73 |
| #4 | (bronchial non small cell cancer):ti,ab,kw OR (bronchial non small cell carcinoma):ti,ab,kw OR (lung non small cell cancer):ti,ab,kw OR (lung non small cell carcinoma):ti,ab,kw OR (non oat cell lung cancer):ti,ab,kw OR (non small cell bronchial cancer):ti,ab,kw OR (non small cell lung cancer):ti,ab,kw OR (Non Small Cell Lung Carcinoma):ti,ab,kw OR (non small cell pulmonary cancer):ti,ab,kw OR (non small cell pulmonary carcinoma):ti,ab,kw OR (non squamous NSCLC):ti,ab,kw OR (non-oat cell lung cancer):ti,ab,kw OR (nonsmall cell carcinoma of the lung):ti,ab,kw OR (Nonsmall Cell Lung Cancer):ti,ab,kw OR (Non-Small Cell Lung Cancer):ti,ab,kw OR (nonsmall cell lung carcinoma):ti,ab,kw OR (Non-Small Cell Lung Carcinoma):ti,ab,kw OR (Non-Small-Cell Lung Carcinoma):ti,ab,kw OR (Non-Small-Cell Lung Carcinomas):ti,ab,kw OR (pulmonary non small cell cancer):ti,ab,kw OR (pulmonary non small cell carcinoma):ti,ab,kw | 16086 |
| #5 | (biologic response modifier therapy):ti,ab,kw OR (biological response modifier therapy):ti,ab,kw OR (BRM therapy):ti,ab,kw OR (immune therapy):ti,ab,kw OR (immunogenic therapy):ti,ab,kw OR (immunoglobulin therapy):ti,ab,kw OR (immunological therapy):ti,ab,kw OR (immunological treatment):ti,ab,kw OR (immunomodulant therapy):ti,ab,kw OR (immunomodulary therapy):ti,ab,kw OR (immunomodulating therapy):ti,ab,kw OR (immunomodulation therapy):ti,ab,kw OR (immunomodulative therapy):ti,ab,kw OR (immunomodulator therapy):ti,ab,kw OR (immunomodulatory intervention):ti,ab,kw OR (immunomodulatory therapy):ti,ab,kw OR (immunomoduling therapy):ti,ab,kw OR (immunomodurating therapy):ti,ab,kw OR (Immunotherapies):ti,ab,kw OR (Immunotherapy):ti,ab,kw | 45168 |
| #6 | (clinical economics):ti,ab,kw OR (dental economics):ti,ab,kw OR (hospital economics):ti,ab,kw OR (medical economics):ti,ab,kw OR (nursing economics):ti,ab,kw OR (health economics):ti,ab,kw | 15438 |
| #7 | (Pembrolizumab):ti,ab,kw OR (Keytruda):ti,ab,kw OR (Nivolumab):ti,ab,kw OR (Cemiplimab):ti,ab,kw OR (Durvalumab):ti,ab,kw OR (Atezolizumab):ti,ab,kw OR (Ipilimumab):ti,ab,kw OR (Camrelizumab):ti,ab,kw OR (Sintilimab):ti,ab,kw OR (Tislelizumab):ti,ab,kw OR (Sugemalimab):ti,ab,kw OR (Serplulimab):ti,ab,kw OR (Tiragolumab):ti,ab,kw OR (Toripalimab):ti,ab,kw OR (Tremelimumab):ti,ab,kw | 8462 |
| #7 | (#1 OR #4) AND (#2 OR #5 OR #7) AND (#3 OR #6) | 13 |

**Search strategy of web of science**

| NO. | Search deatiles | Results |
| --- | --- | --- |
| #1 | (((((((((((((((((((TS=(bronchial non small cell cancer) OR TS=(bronchial non small cell carcinoma)) OR TS=(lung non small cell cancer)) OR TS=(lung non small cell carcinoma)) OR TS=(non oat cell lung cancer)) OR TS=(non small cell bronchial cancer)) OR TS=(non small cell lung cancer)) OR TS=(Non Small Cell Lung Carcinoma)) OR TS=(non small cell pulmonary cancer)) OR TS=(non small cell pulmonary carcinoma)) OR TS=(non squamous NSCLC)) OR TS=(non-oat cell lung cancer)) OR TS=(nonsmall cell carcinoma of the lung)) OR TS=(Nonsmall Cell Lung Cancer)) OR TS=(Non-Small Cell Lung Cancer)) OR TS=(nonsmall cell lung carcinoma)) OR TS=(Non-Small Cell Lung Carcinoma)) OR TS=(Non-Small-Cell Lung Carcinoma)) OR TS=(Non-Small-Cell Lung Carcinomas)) OR TS=(pulmonary non small cell cancer)) OR TS=(pulmonary non small cell carcinoma) | 156990 |
| #2 | ((((((((((((((((((TS=(biologic response modifier therapy) OR TS=(biological response modifier therapy)) OR TS=(BRM therapy)) OR TS=(immune therapy)) OR TS=(immunogenic therapy)) OR TS=(immunoglobulin therapy)) OR TS=(immunological therapy)) OR TS=(immunological treatment)) OR TS=(immunomodulant therapy)) OR TS=(immunomodulary therapy)) OR TS=(immunomodulating therapy)) OR TS=(immunomodulation therapy)) OR TS=(immunomodulative therapy)) OR TS=(immunomodulator therapy)) OR TS=(immunomodulatory intervention)) OR TS=(immunomodulatory therapy)) OR TS=(immunomoduling therapy)) OR TS=(immunomodurating therapy)) OR TS=(Immunotherapies)) OR TS=(Immunotherapy) | 1885286 |
| #3 | ((((TS=(clinical economics) OR TS=(dental economics)) OR TS=(hospital economics)) OR TS=(medical economics)) OR TS=(nursing economics)) OR TS=(health economics) | 548199 |
| #4 | (((((((((((((TS=(Pembrolizumab) OR TS=(Keytruda)) OR TS=(Nivolumab)) OR TS=(Cemiplimab)) OR TS=(Durvalumab)) OR TS=(Atezolizumab)) OR TS=(Ipilimumab)) OR TS=(Camrelizumab)) OR TS=(Sintilimab)) OR TS=(Tislelizumab)) OR TS=(Sugemalimab)) OR TS=(Serplulimab)) OR TS=(Tiragolumab)) OR TS=(Toripalimab)) OR TS=(Tremelimumab) | 47478 |
| #5 | #4 OR #2 | 1903045 |
| #6 | #1 AND #3 AND #5 | 179 |
